# A Qualitative Exploration of Prominent Factors Contributing to the Aetiology of Child and Adolescent Eating Disorder Presentations during the COVID-19 Pandemic: The Perspectives of Patients, Parents and Clinicians

**DOI:** 10.3390/jcm13020615

**Published:** 2024-01-22

**Authors:** Finán Murray, Sharon Houghton, Fay Murphy, Emma Clancy, Dónal Fortune, Fiona McNicholas

**Affiliations:** 1 Department of Psychology, University of Limerick, V94 T9PX Limerick, Ireland; 2 Clinical Psychology Programme, Department of Psychology, University of Limerick, V94 T9PX Limerick, Ireland; sharon.houghton@ul.ie; 3 Lucena Clinic Child & Adolescent Mental Health Services, 59 Orwell Road, Rathgar, D06 HX93 Dublin, Ireland; fay.murphy@sjog.ie (F.M.); fiona.mcnicholas@ucd.ie (F.M.); 4 Children’s Health Ireland Crumlin, D12 N512 Dublin, Ireland; 5 School Medicine & Medical Sciences, University College Dublin, D04 V1W8 Dublin, Ireland

**Keywords:** eating disorder, COVID-19, child and adolescent, aetiology, qualitative, reflexive thematic analysis

## Abstract

Aim: The aim of this study was to explore the prominent factors that contributed to the development of child and adolescent eating disorder presentations during the COVID-19 pandemic. Methods: This was achieved through a qualitative analysis of interviews gathered from (1) adolescent patients diagnosed with an eating disorder (ED) during the COVID-19 pandemic (aged 13–18) (*n* = 4), (2) parents of patients attending an ED service (*n* = 7) and (3) clinicians working within a specialist child and adolescent ED support service (*n* = 7). Reflexive thematic analysis was used to analyse the data and establish the most prominent aetiological factors reported. Results: The most prominent aetiological factors reported were (1) the accumulation of risk factors; (2) seeking control amid increased anxiety, stress and uncertainty; (3) social isolation; (4) an increased focus on exercise and “healthy eating”; (5) increased social media use promoting unhelpful attention towards ED triggers; and (6) a lack of both awareness and support services. Conclusion: During the COVID-19 pandemic, the quantity and severity of ED presentations increased. The current study uncovered six themes perceived by patients, parents and clinical staff that contributed to the aetiology of ED presentations during the COVID-19 pandemic. It is hoped that the insight gained through this research study into ED aetiology may act as a basis for further research and support ED awareness, prevention and intervention in the future.

## 1. Introduction

Internationally, it has been reported that following the onset of the COVID-19 pandemic and associated public health measures, rates of eating disorder presentations increased significantly. From the perspective of patients, their parents and clinicians, this study aimed to identify factors that may have been implicated in the development of child and adolescent eating disorders during the COVID-19 pandemic.

COVID-19 is a global pandemic that emerged in 2019. Due to the spread of the virus through human proximity, many governments imposed public health measures and restrictions intended to limit the physical proximity between people, and thus the spread of the virus. In many cases, these restrictions resulted in significant changes and disruption to elements of lifestyle including social, education, economic, work and leisure activities [1]. A significant increase in mental health symptoms was reported at the early and peak stages of the pandemic; however, among the general population, mental health symptoms were reported to return to pre-pandemic levels by mid-2020, with a continued fluctuation in referral rates reported thereafter [2,3].

However, the extent of the impact of COVID-19 and its associated response measures are not fully understood and concern has been raised within the literature regarding the potential impact on vulnerable populations, and those with pre-existing mental health difficulties [4]. A particularly vulnerable group in the COVID-19 context may be children and adolescents with eating disorders (EDs) [5]. There was an increase in both new and relapsing cases of ED, with increased referrals to both paediatric and adult general hospitals along with community MH services [6]. In Ireland, referrals to child and adolescent mental health services (CAMHS) increased and anecdotal reports by clinicians reported an increase in referral complexity, with a higher proportion reporting suicidal ideation and eating psychopathology [6]. Concern has also been raised regarding the development of new ED presentations, particularly among vulnerable populations [5,7] and presentations of atypical cases [8]. The ED-specific research shows an increase in symptomology and referral rates early in the pandemic [9]. A more recent systematic review [10] concluded that there was a large increase in ED symptoms and hospital admissions increased by 48% over the pandemic. Increases in the number of eating disorder helpline contacts have also occurred [11].

Eating disorder (ED) research has identified several risk factors for disordered eating in the context of COVID-19 including disrupted routines, reduced peer-related activity and increased time on social media, with the possibility of weight-stigmatising messages [12,13,14]. Public health restrictions may have exacerbated barriers to the assessment and treatment of EDs, with longer waiting lists, concerns regarding the presentation of even more acute cases [15,16], limited face-to-face care and changes in the care that families and carers receive [17,18].

Many of the known ED aetiological and perpetuating factors were suggested to be impacted by the COVID-19 pandemic and related [1]. Rodgers et al. (2020) [19] proposed three primary pathways through which the COVID-19 pandemic impacted ED presentations: (1) an increase in ED risk factors, (2) a decrease in the factors that protect against EDs and (3) exacerbated barriers to care.

The emerging research and literature raised significant concerns as to the potential for the COVID-19 pandemic and related experiences to contribute to the aetiology and exacerbation of EDs for many children and adolescents globally. However, our understanding of the nature, quality and extent of the myriad complex pathways through which COVID-19 exerted its impact remains in its infancy. Although COVID-19 is predicted to have severe impacts on EDs, the pathways through which this impact is occurring are less well understood [5]; the perceptions of expert clinicians and experts through experience may have much to offer for increasing the understanding of the key factors contributing to the impact of the COVID-19 pandemic on EDs.

Thus, using reflexive thematic analysis [20] to analyse the perspectives of participants from three groups: (1) adolescents diagnosed with an ED during the COVID-19 pandemic, (2) parents of children and adolescents who were diagnosed with an ED during the COVID-19 pandemic and (3) clinicians working in a specialist child and adolescent ED service during the COVID-19 pandemic, this current study aimed to explore prominent factors that were perceived to have contributed to the aetiology of child and adolescent eating disorder presentations during the COVID-19 pandemic.

## 2. Research Question

What were the most prominent factors identified by young people, parents and clinicians that contributed to the aetiology of child and adolescent eating disorder presentations and diagnoses during the COVID-19 pandemic?

## 3. Methods

### 3.1. Participants

A purposive, non-probability sampling method was used. Recruitment and data collection took place between March and April 2022. Perspectives of patients, their parents and clinicians working within a specialist child and adolescent ED service based in Ireland were sought. The specialist Child and Adolescent Mental Health Services (CAMHS) ED service provides a service for all youth aged under 18 years in a large geographical area of Ireland, totalling 260,560 youth or 12.7% of the youth population in Republic of Ireland.

Three groups of participants were recruited and the inclusion criteria were as follows:

(1) Patients: (*n* = 4) adolescent patients attending an outpatient CAMHS (child and adolescent mental health service) specialist in treating ED, with new onset ED diagnosed during the COVID-19 pandemic (March 2020–April 2022) and deemed clinically suitable to safely participate in this research by the member of their clinical team. Personal demographics were kept to a minimum to protect patient confidentiality. The sample included 3 females and 1 male, all with a diagnosis of anorexia nervosa. The age range was 13–18 years and patients were not related/from the same household.
(2) Parents: (*n* = 7) parents/legal guardians of children and adolescents (aged 10–18) attending the CAMHS ED service and diagnosed with an ED during the COVID-19 pandemic.
(3) Clinicians: (*n* = 7) professional health care practitioners working within the ED specialist CAMHS during the COVID-19 pandemic. This group incorporated a range of health care practitioners: psychiatrists, psychologists, clinical nurse specialists and social workers.

Written consent was obtained prior to all participants partaking in the study. Ethical approval was granted by the University of Limerick Research Ethics Committee and the St John of God Ethics Committee responsible for the clinical ED service.

Thirty-two participants were invited to participate, of whom eighteen agreed to participate, with seven in the parent group, seven in the clinician group and four in the patient group (three female, one male; all four reported a diagnosis of anorexia nervosa). Fourteen participants (one clinician, five parents and eight patients) did not return consent forms and so were not included in the study. Several participants were from the same family: daughter, mother, father; father, mother; mother, daughter; mother, son; mother, daughter.

### 3.2. Data Collection

The data were collected through semi-structured qualitative interviews conducted with participants from the three groups outlined above. Interviews were conducted online via Zoom and all interviews were audio-recorded for later transcription and qualitative analysis.

Primarily, the qualitative interviews adopted an inductive approach and aimed to explore participants’ perspectives on factors that contributed to the exacerbation or aetiology of ED during the COVID-19 pandemic. The flexible interview schedule was developed to gain general insight into the participants’ perspectives on ED aetiology, whilst allowing freedom to focus on prominent issues established by participants as being especially pertinent to the research question.

### 3.3. Data Analysis

Reflexive thematic analysis (RTA) [20,21] was used to establish the prominent themes within the data. RTA was selected as the most appropriate approach to answer the research question. Through RTA, themes and subthemes may be considered deductively and/or inductively and themes may contain manifest or latent content [22]. This approach allowed the analysis to remain flexible and open to establishing prominent and novel constructs within the data whilst also being informed by prior literature and theory relevant to the current research question.

RTA was used to establish the most pertinent elements of the entire data set relative to the research question. This analytic process facilitated the organisation, description and interpretation of the main themes evident within the data [20,21].

The following methods were used to achieve a rigorous reflexive thematic analysis of the data in line with Braun and Clarke [20,21,23,24] (1) The analysis commenced with data immersion and familiarization. This was followed by (2) preliminary coding (2 coders), (3) re-reading the data to develop and adjust the coding and (4) clustering and creation of potential themes based on the codes created. (5) was for team discussion and themes were reviewed by the research team before the last step (6) of writing up, editing and naming the themes and sub-themes, leaving what were deemed to be the most pertinent themes, sub-themes and quotes representative of the data set and relative to the research question.

## 4. Results

This qualitative study explored and integrated participants’ perspectives on factors that contributed to the aetiology of EDs during the COVID-19 pandemic. The following section documents the primary themes and subthemes that were established.

### 4.1. The Accumulation of Risk Factors: “The Perfect Storm”

There was an increase in the number of referrals and the severity of presentations. Clinicians reported a significant increase in the number of referrals and the severity of cases presented during the COVID-19 pandemic period.

“As the pandemic developed, we started seeing a huge increase in the numbers of referrals… I’d say it’s somewhere between three or four times what we would have seen normally” (P6 Clinician).

“Clients are sicker than I’ve ever seen before. Their weight loss seems to have been much faster and the restriction is much more intense” (P2 Clinician).

Generally, participants were not able to identify one specific factor that caused the ED to develop. Instead, it was the intensification and accumulation of multiple risk factors that contributed to the aetiology of ED cases during this time.

“There was something about the perfect storm during the pandemic that was driving these kids or adolescents who probably had a predisposition, or had subclinical ED patterns, causing it to become more pronounced” (P15 Clinician).

### 4.2. Seeking Control Amid Increased Anxiety, Stress and Uncertainty

The increase in uncertainty and anxiety caused by the COVID-19 pandemic contributed to children and adolescents seeking a sense of control through exercise and dieting.

“They needed to control the anxiety… They couldn’t control the whole world and there was no adult in their life that would tell them what was happening exactly, and what the next steps were going to be” (P2 Clinician).

“His way of dealing with it was with controlling the food. He could control what he was eating, and he could control the exercise he was doing. And I think that was a major cause” (P12 Parent).

### 4.3. Change to Routine and Lack of Structure

The abrupt change to routine and lack of structure linked with home-schooling and restrictions to extracurricular activities was suggested to be a contributing factor.

“Young people really struggled with there being no daily routine with home-schooling. Things were a bit all over the place, and they needed structure. They wanted structure. And they wanted control… they felt out of control and they needed to do something and didn’t know what to do. So they just stopped eating” (P2 Clinician).

### 4.4. School Closures and the Transition to Home-Schooling

In addition to changes in routine, all participants reported that school closures posed academic and learning problems for young people, who in many cases struggled to apply themselves to the discipline of home-schooling. Frustrations and time unoccupied were perceived to contribute to an overly large focus on body image, and for a wish to return to school in person.

“When we all went off school and we had nothing to do, we were just at home…I thought it was just really boring. And it was very hard to learn stuff and we were just sitting there for hours. I didn’t like it at all… That’s when it started. I didn’t ever think about what I looked like or what I should be eating before that… I’d rather be in school, you know, doing everything normally again” (P3 Client).

### 4.5. Perfectionistic Tendencies Coupled with a Lack of Focus and Sense of Achievement

Among young people with tendencies toward being highly perfectionistic and achievement-focussed, restrictions on outlets, such as school, extracurricular activities and sports, were replaced by extreme home exercising and dieting.

“Temperamentally, they would have always had an anxious temperament, wanted to achieve, very into sport, very competitive, always wanted to push themselves. But it was always very much in a structured environment. Something that actually tipped them over, was the falling away of structures… When that fell away, when they weren’t getting that sense of themselves through achieving… I think the school and the sporting activities were that healthy environment that helped them to not bring it to those extremes” (P15 Clinician).

The adolescent clients described how increased motivation resulted in their capacity to persist with diet and exercise regimes.

“I stick to something, you know, until I finish it. Some of my friends, they would do the same thing, cut down on their eating, but then the next day, they wouldn’t be able to finish it, but I would keep going” (P14 Client).

### 4.6. Increased Stress in the Family Home

Long-term confinement to the family home due to COVID-19 restrictions with limited external outlets resulted in increased stress and challenged family relationships, and thus was a contributing factor to ED aetiology.

“Family dynamics changed so much. They were there all the time. Family weren’t the people they could go to and talk about their problems anymore, because they were becoming the problem. The fighting at home, the increased tetchiness with each other, just being around each other all of the time” (P2 Clinician).

Additionally, pre-existing parental stressors such as homeworking and financial pressures were suggested to contribute to the overall burden of stress on young people during this time.

“Parents are coming home increasingly stressed because of issues around employment or housing and cost of living…and the kids can tell they are stressed…so I’m not sure if the COVID-19 pandemic was the cause. I think it was a catalyst. I think the underlying stuff was actually the cause, but the sea-wall, or whatever held back the tide, broke during the pandemic” (P6 Clinician).

### 4.7. Parents Working on the Frontline

For young people whose parents were frontline workers during the pandemic, the increased work hours and associated increased stress and anxiety were suggested to be a further contributing factor.

“We had a cohort of children whose parents were on the frontline. That was a factor…the increased stress level and anxiety at home because they had parents on the frontline” (P7 Clinician).

### 4.8. Social Isolation

Social distancing restrictions preventing young people from meeting friends and family contributed to social isolation, which in turn contributed to the ED aetiology during the pandemic period.

“The isolation is probably the biggest factor, you know. She didn’t see her friends, we didn’t socialize. So there was just a disconnect…there was isolation from family and friends” (P4 Parent).

### 4.9. COVID-19-Related Anxiety and Vulnerable Family Members

Fears of contracting the COVID-19 virus and transmitting it to vulnerable family members contributed to increased anxiety within family homes and further increased social isolation.

“The stress of trying not to catch COVID, especially me because I had someone who was high risk at home. And so I was really stressed. I couldn’t go out anywhere, I couldn’t meet any of my friends, because I didn’t want to get it and I didn’t want to bring it home. So I think the stress of that definitely was a big factor in contributing to the eating disorder” (P3 Client).

### 4.10. Increased Focus on Exercise and Diet

The increased focus on exercising and eating a healthy diet that was prevalent among the general population in Ireland during the pandemic period was taken to extremes by these young people and contributed to the aetiology of EDs. The lack of focus, structure, routine and boredom triggered this focus on diet and exercising initially.

“It actually started with over-exercising. And when I started exercising, I just got more obsessive and obsessive. I just started over exercising, and then my food started to be cut down over time as well. That went on for about a year or something. And then it just got worse and worse… everyone was trying to use this opportunity to get fit and everything, but I just took that to an absolute extreme” (P3 Client).

Clinicians reported an increased focus on exercise and fitness among clients presenting with an ED during the pandemic period.

“That is the new kind of manifestation of EDs, it’s not always about the thinness, it’s about the fitness, and we see that a lot” (P15 Clinician).

In combination with this increased exercise and fitness focus, participants described an increased focus on diet. Young people were reportedly motivated to eat ‘healthy’ foods; however, there was a lack of understanding of the necessity to consume enough calories to replenish those used through exercise. Diets were taken to extremes and important factors such as maintaining a balanced diet and a healthy relationship with food were lost. These themes of exercise and healthy eating were reportedly encouraged initially by parents, health officials, and general media, but they were taken to extremes by children with pre-existing vulnerabilities.

“A lot of the patients are talking about this healthy eating. They keep using the term healthy eating. But when you listen to what they describe as healthy eating; it’s dieting. So what they’re being taught is healthy eating, isn’t healthy eating. It’s not balanced” (P2 Clinician).

Clinicians warned of the dangers surrounding messages in media related to exercise and dieting and suggested that although “there is a huge problem with obesity…the one size fits all message doesn’t fit all. And you need to be really mindful of the young people who don’t need that message” (P10).

### 4.11. Increased Social Media Usage Perpetuated by Algorithms

Participants reported that screen time and social media use increased during the pandemic.

“I spent a lot of time on Tiktok. I was just always scrolling on the videos on Tiktok. That kind of became an addiction because of lockdown. I was on my screen, including school, probably 16 h a day of non-stop looking at a screen” (P17 client).

“She wouldn’t have been that interested before. She was just too busy, with schoolwork and music and sports and whatever she was doing. But because she was at home so much, of course there was more engaging with social media” (P4 Parent).

This finding was consistent with the literature base which suggested that, among adolescents, social media usage and screen-time have gradually increased in recent years but spiked during the COVID-19 pandemic [25,26,27].

The algorithms used by social media platforms which target users with content aligned with what they viewed previously, meant these young people were predominantly exposed to media related to exercise, food and body image through their social media platforms.

“I just think, social media was really bad at the very beginning. For me, and I think for a lot of other young people who I’ve talked to, because the social media platforms, kind of create, a user experience that is customised to what you look at. So my social media platforms were all focused around eating disorders… If you got hooked on what we called, eating disorder TikTok, it creates your own page. If you have an eating disorder, or if you’re struggling with, eating disorder thoughts, it focuses in on it. I find that so unhelpful, because it was all just about people showing off their bodies and people sharing what they ate; which was a tiny amount. And then I thought, Oh, my God, if they’re eating so little, I have to eat less than that. So I think that was just really, really unhelpful” (P3 Client).

### 4.12. Social Media Promoted Unrealistic Perceptions of Body Image

Young people were bombarded with unrealistic examples of the ‘ideal’ body image via social media. This contributed to a distorted perception of normal body shape, and thus contributed to the development of EDs.

“The bombardment from social media. They spend hours scrolling up and down. Looking at pictures of other teenagers and what they look like, their body shapes, photographs of the ideal looking girl or boy. It’s a bombardment. And if you look at it often enough, you come to believe that that is the way you’re supposed to be” (P7 Clinician).

In addition to this, young people felt pressure regarding how they portrayed themselves on social media, which further contributed to body image scrutiny and insecurity.

“They couldn’t physically see their friends but they had to interact with them on social media and share pictures, so that pressure was much higher. Because you take the picture and then you look at it before you send it. So there was much more scrutinization of themselves. There were 50 pictures being taken before one was actually sent” (P2 Clinician).

“People have to be perfect on social media. You don’t get to be yourself on it. Really.” (P17 Client).

### 4.13. Social Media Promoted Excessive Exercise and Dieting

Trends on social media promoted extreme exercising and dieting. The regular viewing of this material resulted in young people engaging in exercise and diet behaviours at excessive and dangerous levels.

“On social media, there were lots of challenges concerning weight loss, exercise, and healthy eating. It was about being able to wear a bikini, and have abbs, and be really toned and low fat percentile and high muscle ratios. All this stuff being put in their face every day while scrolling through Instagram. A lot of them started following fitness pages and fitness bloggers and food pages. A lot of pro-anorexia stuff without realising what it was” (P1 Clinician).

### 4.14. Lack of Awareness and Support Services

During initial encounters with general healthcare services, some participants described encountering a lack of awareness and understanding of EDs among healthcare professionals and felt concerns regarding EDs were at times not treated appropriately. This prevented early detection and acted as a barrier to support. In addition to this, parents reported that a lack of services was also prohibitive to care.

“We couldn’t get her seen by services. They were so overrun…We were looking at her wasting away from one week to the next. And we didn’t have anywhere to go” (P4 Parent).

A lack of awareness among parents and a reluctance to act was also reported to contribute to cases of EDs going unnoticed for longer.

“I suppose the truth is, we didn’t really recognize that there was any problem. You know, we weren’t looking out for it, her behaviour in terms of eating…we do feel some guilt because we didn’t spot it early enough, and might have got her help sooner” (P5 Parent).

## 5. Discussion

Through in-depth qualitative interviews and analysis, conducted with participants with significant experience of, or insight into an ED during the COVID-19 pandemic (clients, parents and clinicians), this study provided novel insights into the prominent aetiological factors perceived to have contributed to the development of EDs among this cohort during this timeframe. The qualitative methodology and reflexive thematic analysis used in this study allowed flexibility and facilitated the aetiological factors deemed most pertinent by participants to be identified [20,21].

A number of key themes were identified in this study. The finding that the overall accumulation of risk factors led to an increased number and severity of presentations is consistent with the wider literature base, which suggests that the aetiology of EDs occurs through a complex interplay of unique biological, psychological and social factors [28]. As a result of this accumulation and intensification of risk factors, the clinicians interviewed reported observing a significant increase in the prevalence and severity of symptoms among ED cases presenting during the COVID-19 pandemic. This concern is supported by emerging research globally; however, additional research is needed to accurately evaluate the extent of this exacerbation [9,15].

The novelty and associated unexpectedness of COVID-19, including the fluctuating public health restrictions, might be considered to have contributed to the sense of a lack of personal control and was reflected in the theme ‘seeking control amid increased anxiety, stress and uncertainty’. The change of routine and lack of structure leading adolescents to seek control through exercise and dieting emerged as a theme, one which has been echoed in other qualitative studies of the impact of the pandemic on ED symptomatology in adult populations [10].

All participants in the current study reported the school closures as a significant source of difficulty for young people with anorexia. School closures were highlighted by world leading experts at the early stages of the pandemic as an important focus for research [29]. In addition, pandemic-related school closures have since been linked to higher levels of health complaints and severe or moderate anxiety in young people [30].

The current study found that the removal of positive outlets for their achievement drive, due to COVID-19 restrictions, precipitated EDs among the child and adolescent cohort through a disrupted sense of identity, increased internalisation of psychological distress and the development of new goals focused on extreme exercise and weight loss. The literature indicates that perfectionistic tendencies and high motivation levels and achievement focus are common personality traits shared by the adolescent ED cohort [31,32], and that perfectionism combined with high achievement focus and motivation can create a sense of control and contribute to the adolescent’s sense of identity [33].

That families were under increased stress during the pandemic has been established [34] and this was reflected in the current study when increased stress and strained family relationships was linked to the ED aetiology, with a specific focus on families in which the parents were frontline workers.

Social connection is a key component in social and emotional development for young people at all stages of development. The absence of social connection was reported to increase vulnerability to mental health difficulties and EDs [12]). In addition, in this study, social isolation was reportedly exacerbated among families where there was someone with an increased vulnerability due to a compromising health condition living in the home. These restrictions to social contact had further negative consequences, as important sources of support, both for the young people and their parents, were significantly limited.

Participants reported that the COVID-19 environment acted as a trigger for initiating exercise and eating patterns that were later developed to excessive levels and contributed to the development of EDs. During COVID-19, and in an effort to try and encourage healthy eating and physical activity during lockdown, media messaging focused on increasing physical activity, following healthy meal plans and cooking and eating together. Whilst well intentioned, it is clear that these messages may have ‘triggered’ the more vulnerable to become overly preoccupied with a fitness regime. A recent systematic review examining the potential associations of anti-obesity public health messages and disordered eating found some evidence that public health messages were stigmatizing towards individuals with high BMIs, exacerbating thin ideals and the drive for thinness, but not body dissatisfaction (Bristow, Meurer, Simmonds & Snell, 2020) [35]. Messages encouraging reduced meal portions were viewed as potential triggers for disordered eating In the current study. To what extent the increase in ED presentations was due to overzealous public health messaging in groups vulnerable to developing EDs remains unknown.

In this study, an increased focus on exercise and diet was attributed to the increased time spent at home, the lack of structure and routine linked with restrictions to school, and extracurricular activities which contributed to boredom, alongside the increase in social media use, which intensely promoted extremes of exercise and diet. The stay-at-home orders and the ensuing perception of ‘social isolation’ removed the usual protective mood enhancing and interpersonal focus found with social connectedness. Given the salience of social contact and peer relationships during childhood, it is understandable how school closures and reduced social contact might have contributed to youth distress [30].

Throughout the current study, the risk factors associated with social media usage were suggested to be multi-faceted and cumulative, and the algorithms used by social media platforms were suggested to perpetuate compulsive usage. Participants described how young people were “constantly bombarded” with unrealistic body image, extreme exercise and fitness influences, and extreme and dangerous diet regimes. The regular exposure to media promoting extremes of body image, exercise and diet was reportedly a significant factor contributing to the development of ED behaviours and cognitions and later the development of EDs among young people; these findings are supported by other recent research [26,36]. The impact of social media use and importance of clinicians checking in with their ED patients regarding social media use have been highlighted [37,38]; however, the prominence of social media algorithms in this current research suggests that checking in with clients might need to be supplemented by work with social media platforms.

The closure of many services in an effort to contain the spread of COVID-19 may have contributed to the identified theme of a ‘lack of awareness and support services’, suggesting that there were difficulties obtaining the appropriate information and access to the appropriate services. The respondents reported that the situation was exacerbated by the lack of resources and support services available. When potential EDs were identified, long waiting times and difficulties accessing support services acted as a significant barrier to support, hindered recovery and greatly added to stress within the family system. These findings correlate with recent findings from the Irish context which describe long waiting times, significantly under-resourced support services, significant barriers to care and a need for reform [39]. In addition to this, Steinglass and Walsh (2016) [40] highlighted the importance of early diagnosis and treatment for EDs and its impact on positive outcomes for clients.

An adaptation to clinical delivery and the uptake of telepsychiatry by services within the organization were put in place [18], but this research suggests that, despite these measures, parents experienced a lack of support. Adults also experienced adverse effects linked to reduced access to services [41], but these may be of particular concern to parents of youth with anorexia nervosa, given the recognized high rates of medical complications seen in this population [42]. Higher rates of lowered immunity and peripheral circulatory problems in nutritionally compromised patients with AN may have had particular salience during the pandemic period. The disruption to clinical services in COVID-19 is likely to have exacerbated the established high levels of caregiver burden that were present among parents of youth with eating disorders [43].

Our findings also align with other qualitative studies which have highlighted similar adverse aspects following the COVID-19 public health restrictions. In a study among UK adults with EDs, the core themes identified were similar to those in our study. These included social restrictions, functional restrictions (which included a lack of routine and structure) and restrictions in access to services. This study also found some positive aspects which were associated with ‘a drive for recovery’ [1]. A recent systematic review, which included eight qualitative and six mixed-methods studies, reported on similar themes to those identified in our study [10]. Changes to routine, loss of structure, loss of control, triggering media messaging and isolation were all themes identified as contributing in a negative way towards new onset eating behaviours or psychopathology. Of interest in this systematic review, the authors also noted a number of positive aspects during the pandemic that were not reported in our study. These included the beneficial impact of being shielded during lockdown from previous stressors or triggers, using free time for self-care or self-reflection, and contrary to the sense of social isolation reported by our cohort, a perceived increase in social support [10]. This highlights the importance of understanding the nuanced and individual responses caused by stressors in different cohorts.

The inclusion of adolescent participants with EDs recently diagnosed during the COVID-19 pandemic is a significant strength of this study. The inclusion of parents and clinicians further bolstered the participant sample and helped provided more comprehensive insights into the research question.

However, the small and relatively homogeneous sample, recruited from one ED support service in a geographic location in Ireland, and with multiple participants from the same family, may mean the findings are not generalizable. However, the specialist Child and Adolescent Mental Health Services (CAMHS) ED service provides a service for all youth aged under 18 year in a large geographical area of Ireland, totalling 260,560 youths or 12.7% of the youth population in Republic of Ireland. 

The small sample prevented subgroup analysis across variables such as diagnoses, gender or age, which may have provided additional insight into ED aetiology commonalities and differences across different cohorts. Further research and replication will be important to increase the validity of the current findings and continue to enhance the understanding of the aetiological factors that contribute to ED development.

## 6. Conclusions

Based on the current research, the following factors were prominent in the aetiology or exacerbation of EDs during the COVID-19 pandemic: (1) the accumulation of risk factors; (2) seeking control amid increased anxiety, stress and uncertainty; (3) social isolation; (4) an increased focus on exercise and “healthy eating”; (5) increased social media use promoting unhelpful attention towards ED triggers; and (6) a lack of awareness and support services. These themes align with some other qualitative studies that have been conducted in other countries. It is hoped that the insight into ED aetiology gained through this study may act as a basis for further research, and support ED awareness, prevention and intervention in the future. The need to have responsive services and clear pathways to diagnosis and treatment, particularly during a public health emergency, was highlighted in the current study. This research highlights the need in clinical practice not only to check in with young people around social media use, but also to alter social media to halt the potential exacerbation of their problems through excessive exposure to particular content. Raising awareness of this phenomenon with social media platforms will be an important part of reducing potential harm. In the case of any future need for public health measures involving school closures and restricted social contact, the impact thereof on young people’s mental health must be carefully considered and limited to those strictly necessary. In the event of future pandemics, consideration of the impact of the ‘perfect storm’ on the development of EDs should be central to public health decision making.

## Data Availability

The datasets presented in this article are not readily available, because the ethics process under which the research was conducted stipulated that data be managed in a highly secure manner, so as to protect participants confidentiality. Permission was not given for the entire data set to be openly published. The ethics document stipulated that only anonymous extracts from the data would be shared in the research paper. Requests to access the dataset should be directed to Dr Finan Murray.
